# The effects of cell therapy on seizures in animal models of epilepsy: protocol for systematic review and meta-analysis of preclinical studies

**DOI:** 10.1186/s13643-019-1169-3

**Published:** 2019-11-01

**Authors:** Naomi Dvir, Muhammad S. Javaid, Nigel C. Jones, Kim L. Powell, Patrick Kwan, Terence J. O’Brien, Ana Antonic-Baker

**Affiliations:** 1 The Department of Neuroscience, Central Clinical School, Monash University, Melbourne, Australia; 2 Department of Medicine, Royal Melbourne Hospital, The University of Melbourne, Parkville, Victoria Australia; 3 Department of Neurology, Alfred Health, Melbourne, Victoria Australia

**Keywords:** Epilepsy, Stem cells, Cell therapy, Animal models

## Abstract

**Background:**

Epilepsy is one of the most common and serious brain conditions, characterised by recurrent unprovoked seizures. It affects about 1% of the population worldwide. Despite a range of antiepileptic drugs being available, one third of the patients do not achieve adequate seizure control. Only a minority of these patients may be suitable to undergo surgical resection of the seizure focus, but this is an invasive and not always successful procedure. There is an urgent need to develop more effective treatment options for uncontrolled seizures. With the recent advances in regenerative and translational medicine, cell therapies could prove to be beneficial. Here we describe the protocol for a proposed systematic review and meta-analysis to assess the effects for cell transplantation in animal models of epilepsy.

**Methods:**

We will include all preclinical animal models of epilepsy that evaluate the effects of cell transplantation compared to the untreated control. The primary outcome will be the change in frequency and duration of seizures from baseline measured by video electroencephalography (EEG). The secondary outcomes will include histological and neurobehavioural assessments. We will perform an electronic search of MEDLINE via PubMed, Web of Science, and EMBASE. Search results will be screened independently by two reviewers and confirmed by a third reviewer. Data from eligible studies will be extracted and pooled, and the summary estimate of effect size will be calculated using DerSimonian and Laird random effects meta-analysis. Heterogeneity will be explored using sub-group meta-analysis, and meta-regression risk of bias will be assessed by using the CAMARADES checklist for study quality tool.

**Discussion:**

The purpose of this systematic review is to assess and summarise the existing literature in the field of cell transplantation as a treatment for epilepsy in animal models. Efficacy will be measured by evaluating the reduction in seizure intervals, number, and duration, within animal models of epilepsy. Analysis of the existing literature will mark the achievement made in the field and locate the existing gaps, a process that will aid in the search for the next needed step.

**Systematic review registration:**

CRD42018103628

## Background

Epilepsy is one of the most common and serious neurological conditions, affecting over 50 million people worldwide [1]. The incidence of epilepsy varies with age, but shows a bimodal distribution with the highest rates seen in early childhood and in adults over 65 years of age [2]. In the majority of cases, seizures are controlled by the administration of one (monotherapy) or a combination of anti-epileptic drugs (polytherapy). Unfortunately, for about a third of the patients, persistent seizures occur despite drug treatment [3]. Further, all current anti-epileptic drugs work by reducing the chance of seizure occurrence without modifying the underlying biological mechanisms of seizure generation [4, 5]. Hence, a major focus of translational research in the epilepsy field has been on developing “disease-modifying therapies”.

Recent advances in regenerative and translational medicine have provided an opportunity to assess whether stem cell therapies could prove to be beneficial for the treatment of epilepsy. A number of clinical trials have assessed the effects of stem cell therapy in stroke [6–9] and Parkinson’s disease [10]. In epilepsy, the efficacy of stem cell therapy has not yet been clinically evaluated. Before this therapy can be translated into the clinic, we need to assess the quality of the pre-clinical research. Here we report a protocol that outlines steps that will be taken to perform a systematic review and meta-analysis to assess the effects of stem cell therapy on seizure in animal models of epilepsy. The objectives of this proposed systematic review and meta-analysis will be (i) to establish a summary estimate of the efficacy of cells in animal models of epilepsy, (ii) to ascertain the conditions under which animal experiments demonstrate greatest efficacy, and (iii) to determine any effect of study quality on reported efficacy.

## Methods/design

### Protocol and registration

This protocol was written in accordance with the Preferred Reporting Items for Systematic Reviews and Meta-Analysis Protocols (PRISMA-P) guidelines [11] through a discussion with our scientific research team that comprises of clinicians (PK and TOB) and translational scientists (ND, MSJ, KLP, NCJ, and AAB). This protocol is registered in PROSPERO (CRD42018103628).

### Disease of interest

Epilepsy is characterised by recurrent unprovoked seizures, resulting from excessive electrical discharges in a group of neurons that fire in synchrony. It has been reported to be caused by a disruption in the balance between neural excitation and inhibition in the brain [12]. There are a number of different animal models of epilepsy that mimic pathophysiological features of the human disease [13]. Among them are chemically induced models such as kainic acid post-status epilepticus model [14], electrical stimulation models [15], kindling models [16, 17], post-traumatic epilepsy models as a result of traumatic brain injury, stroke and brain infections [18], and genetic models [19].

### Intervention assessed

The intervention assessed will be cell transplantation as an epilepsy therapy. The implanted cells will consist of human cells and stem cells and primary embryonic rat or mouse brain cells. Stem cells are characterised as pluripotent cells that can differentiate into different neuronal populations. In this study, we will include stem cell, differentiated neuronal or glial cells with or without genetic manipulation, and mature cells implanted as a repaired tissue.

### Control populations

Cell-based therapies will be compared to untreated or vehicle-treated controls. Only studies that have well-defined controls will be included in this review.

### The outcome measures

Primary outcome measure: video EEG to asses changes in seizures intervals.

Secondary outcome measure: histology and neurobehavioural methods. Neurobehavioural methods such as modified Neurological Severity Scores (mNSS), Morris water maze, Novel Object Recognition Test (NORT) and others in addition to histology methods will be assessed.

### Literature search

We will search three electronic databases: PubMed, Embase, and Web of Science. The search terms to be used to identify the relevant articles are listed below.

For epilepsy: (“Epilepsy” or “Temporal lobe epilepsy” or “TLE” or “seizure” or “epileptogenesis” or “spontaneous recurrent seizures” or “SRS” or “Genetic Generalised Epilepsy” or “GGE” or “frontal lobe epilepsy” or “FLE” or “Photosensitive epilepsy”)

For stem cells: (“stem cells” or “stem” or “haematopoietic” or “mesenchymal”)

For cell therapy: (“cell therapy” or “cell transplantation”)

#### Other sources

To make sure we include all the relevant studies, we will also analyse reference lists of previously published reviews. In addition, the reference lists of included studies will also be reviewed.

The three separate searches will be combined with AND link. All the relevant articles will be combined into single endnote file to remove the duplicates.

### Selection criteria

#### Inclusion criteria

Controlled study comparing outcome in a group of epileptic animals receiving cell-based therapy with a group of animals not receiving the intervention. Transgenic models are included.

#### Exclusion criteria

Studies will be excluded if there is no control group (untreated animals), if the intervention is not cell-based therapy, if the model used is not representative of epilepsy and human studies, and if studies are reviews and editorials.

All included studies will be screened first by title and abstract, followed by full text. The screening will be performed by two independent reviewers (ND and MSJ). All conflicts will be resolved by a third reviewer (AAB).

### Data collection

Data will be extracted by two independent reviewers (ND and MSJ) using pre-approved standardised format. Data will be extracted from text and tables and graphs using digital pixel ruler when no other description is available. We will extract mean ± variance (either standard deviation or standard error of the mean) from each cohort exposed to the intervention (control or cell-based therapy). Cell-based therapy will be categorised as autologous (cells derived from the same animal that is receiving the treatment) or allogeneic (cell derived from a different animal). In addition to the outcome measures, the following information will also be extracted DOI, authors, corresponding author, journal, publication year, and source of funding.

### Risk of bias assessment

Risk of bias will be assessed by the modified CAMARADES risk of bias tool [20]. The tool box was derived for the assessment of risk of bias in animal models of stroke. It includes ten categories: peer-reviewed publication, statement of control of temperature, random allocation to treatment or control, blinded induction of ischemia, blinded assessment of outcome, use of anaesthetic without significant intrinsic neuroprotective activity, appropriate animal model (aged, diabetic, or hypertensive), sample size calculation, compliance with animal welfare regulations, and statement of potential conflict of interests. As the use of anaesthetic without significant intrinsic neuroprotective activity and appropriate animal model (aged, diabetic, or hypertensive) is not relevant for animal epilepsy models, they will be omitted from the risk of bias assessment. For each included study, one point will be given for each of the categories described. Studies with larger scores will be classified as low risk.

### Data analysis

For each individual comparison, we will calculate a normalised effect size as a percentage improvement/or worsening of outcome in the treatment group compared to the control. We will then use DerSimonian and Laird random effects weighted mean difference meta-analysis to calculate a summary estimate of effect size. The data will be presented as percentage improvement in outcome and its 95% confidence intervals [21]. Statistical heterogeneity of included studies will be measured with *I*^2^.

Extent to which the study design characteristics explain differences between studies, we will use sub-group meta-analysis and meta-regression using STATA, and the significance will be set at < 0.05. The subgroup of primary outcome (change in seizure duration and frequency as measured by EEG) will be based on animal species/strain/age, induction of epilepsy, cell tissue source, cell type, route of administration, time of administration/assessment, dose, and cell modification.

To assess the evidence of the publication bias, we will use funnel plot, Egger regression, and trim and fill [22].

## Discussion

Systematic review and meta-analysis are useful tools to quantitatively asses the existing literature and locate existing gaps and controversies in a specific research field.

To date, a number of literature reviews describing stem cell therapies for treatment of epilepsy have been conducted [23–28], but none have been performed in a systematic way or have used meta-analysis to evaluate the overall findings.

This review will summarise all the available data on cell transplantation in animal models of epilepsy. We will report our findings in accordance with the PRISMA statement, and the risk of bias assessment will be performed using the modified CAMARADES risk assessment tool. The objective of this review is to provide valuable insight into the preclinical literature in hope that it will illustrate the gaps in the field which can be addressed by future research. The strength of this review lies within its ability to retrieve and analyse data from different methods of cell culturing and transplantation as well as the inclusion of all epilepsy models to enable a wide-scale analysis.

By performing meta-analysis and meta-regression, we will be able to identify which study characteristics contribute to the variability in the finding which will help in developing more precise preclinical experiments that will pave a path to a successful clinical trial.

To our knowledge, this systematic review is the first of its kind in the field of epilepsy and it builds on already published systematic reviews which assessed the effects of stem cell transplantation in animal models of spinal cord injury [29] and stroke [30].

## Data Availability

Not applicable

## Abbreviations

EEG: Electroencephalography
FLE: Frontal lobe epilepsy
GGE: Genetic generalised epilepsy
mNSS: Modified Neurological Severity Scores
NORT: Novel Object Recognition Test
TLE: Temporal lobe epilepsy
